# Extremely low seasonal prey capture efficiency in a deep-diving whale, the narwhal

**DOI:** 10.1098/rsbl.2022.0423

**Published:** 2023-02-22

**Authors:** Philippine Chambault, Susanna B. Blackwell, Mads Peter Heide-Jørgensen

**Affiliations:** ^1^ Greenland Institute of Natural Resources, Strandgade 91, 2, DK-1401 Copenhagen, Denmark; ^2^ Department of Ecology and Evolutionary Biology, University of California, Santa Cruz, CA, USA; ^3^ Institute of Marine Sciences, University of California, Santa Cruz, CA, USA; ^4^ Greeneridge Sciences Inc., Santa Barbara, CA, USA

**Keywords:** echolocation, acoustics, buzzes, stomach temperature pill, foraging behaviour, prey catch attempts

## Abstract

Successful foraging is essential for individuals to maintain the positive energy balance required for survival and reproduction. Yet, prey capture efficiency is poorly documented in marine apex predators, especially deep-diving mammals. We deployed acoustic tags and stomach temperature pills in summer to collect concurrent information on presumed foraging activity (through buzz detection) and successful prey captures (through drops in stomach temperature), providing estimates of feeding efficiency in narwhals. Compared to the daily number of buzzes (707 ± 368), the daily rate of feeding events was particularly low in summer (19.8 ± 8.9) and only 8–14% of the foraging dives were successful (i.e. with a detectable prey capture). This extremely low success rate resulted in a very low daily food consumption rate (less than 0.5% of body mass), suggesting that narwhals rely on body reserves accumulated in winter to sustain year-round activities. The expected changes or disappearance of their wintering habitats in response to climate change may therefore have severe fitness consequences for narwhal populations.

## 1. Introduction

Successful foraging is essential for individuals to maintain the positive energy balance required for survival and reproduction [1]. Prey capture efficiency is however poorly documented in marine apex predators, despite their crucial role in shaping the food web [2]. Even moderate changes in the abundance and distribution of prey can cause nutritional stress to marine mammals [3], and such cascading effects are amplified in the Arctic due to climate change, compromising the fitness of already sensitive species [4]. Among the three endemic cetaceans living in the Arctic, the narwhal has been recognized as the most sensitive species to climate change [5] due to its reliance on cold Arctic water (less than 2°C), its limited range, complex population structure, specialized diet and strong site fidelity [4,6]. Yet, the seasonal feeding success of narwhals is still unknown largely due to their cryptic behaviour. Despite their extreme philopatry to summer and winter grounds, the function of these habitats in narwhal life history is poorly understood.

Like other echolocating whales [7], narwhals provide information on their foraging activity through the production of buzzes (i.e. prey capture attempts), and an intense foraging effort of up to 250 buzzes per hour [8] has been recorded in narwhals in Scoresby Sound (eastern Greenland) during summer. However, the low feeding activity detected for this population during the same period (7.9 to 17.3 prey captures day^−1^ [9]) contradicts this high foraging effort, suggesting a lowered summer feeding rate, or a scattered or even scarce prey field. To date, foraging effort and feeding activity have only been collected asynchronously on different animals, precluding the inference of prey capture efficiency in this deep-diving cetacean. Yet, estimating the prey capture efficiency of this Arctic predator is needed to assess its vulnerability to climate-induced habitat changes, including potential shifts in abundance and distribution of prey [10].

To improve our understanding of narwhal feeding efficiency, 14 narwhals from the population of Scoresby Sound were instrumented with stomach temperature (ST) pills (STPs), while three of these individuals were simultaneously equipped with acoustic tags. The data collected provided a unique opportunity to relate foraging activity (through buzz detection from acoustic tags) to the capture events (through drops in ST), thereby allowing an estimate of prey capture efficiency and a more accurate summer feeding rate in this deep-diving predator. The low resolution of the STPs (1–2 min) together with substantial data gaps could partially explain the very low feeding rate previously observed in narwhals. The simultaneous use of acoustic tags at a high and continuous sampling rate (1 s) therefore provides a unique tool to correct for the daily food consumption in this species.

## 2. Material and methods

### (a) Animal instrumentation

Between summer 2012 and summer 2016, live-capture operations of narwhals were conducted in collaboration with Inuit hunters in Scoresby Sound fjord, eastern Greenland. Following Heide-Jørgensen *et al.*'s method [9], 14 narwhals were instrumented with STPs, while three of these individuals were simultaneously equipped with acoustic tags (Acousonde) as described in Blackwell *et al*. [8]. From the Acousondes, two variables from that analysis are of interest here: animal depth (every second), and the start time of terminal buzzes, which are believed to indicate prey capture attempts (i.e. foraging dives) [8].

### (b) Detection of prey capture events

The STPs provided by Wildlife Computers had the same properties as the ones deployed in 2014 by Heide-Jørgensen *et al.* [9]. Approximately every 10 s, the STP transmits its temperature to the satellite tag located on the whale's back, but as the satellite tag is very rarely retrieved, only summarized data are transmitted via the Argos system on a 1–2 min sampling rate (see details in the electronic supplementary material, Methods). Examination of the acoustic data collected by the Acousondes revealed that these transmissions produced an identifiable acoustic signal consisting of four pulses with variable inter-pulse intervals (IPIs), which together encoded the ST (electronic supplementary material, figure S1). Considering our goal of comparing buzzing rates (foraging) with successful prey captures (feeding), the analysis of ST signals in the Acousonde records allowed us to increase the data resolution (STP: every minute versus Acousonde: every second) in addition to avoiding possible temporal lag between the two devices. A generalized additive model (GAM) was performed to relate the probability of a drop in ST (feeding probability) to the number of buzzes per dive (foraging probability, electronic supplementary material, Methods).

Due to the tags’ differing sampling resolution, daily STP-based feeding rates were corrected. Based on previous stomach content analyses [11,12], we assumed the main prey of narwhals was the squid *Gonatus fabricii*. The daily food consumption of each narwhal in summer was then estimated based on an average mass of 150 g per prey item ingested [13]. See electronic supplementary material for details on the analyses.

## 3. Results

### (a) Buzz versus stomach temperature drop depth

**Figure 1.**
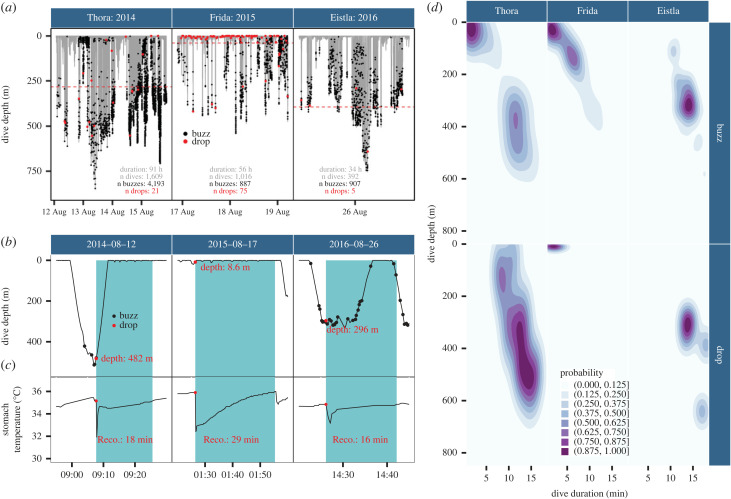
(*a*) Dive profiles over time for the three narwhals equipped with both an STP tag and an Acousonde. Buzzes (foraging attempts) are shown as black dots and ST drops (presumably associated with prey capture events) as red dots. The duration of tracking and the total number of ST drops and buzzes are indicated in each individual panel. The horizontal red lines refer to the mean depth of ST drops for each individual. (*b*,*c*) Zoomed time-series data showing the presumed foraging activity (buzzes) and prey capture events (ST drops) with the dive profiles and (*c*) the ST trends over time. The depth associated with each of the prey capture events is indicated in (*b*) while the recovery time (duration of the events) is indicated in (*c*). Light-blue areas represent the duration of each feeding event from the drop in ST to the end of recovery. (*d*) Density distributions of the depth at which buzzes (top) and ST drops (bottom) occur as a function of dive duration (in min) for each narwhal equipped with both an STP tag and an Acousonde. The colour bar refers to the probability of a buzz or ST drop.

The analysis of 891 h of recordings revealed a total of 458 prey capture events estimated from a drop in ST, while 5987 buzzes were identified for the three whales instrumented with both a STP tag and an Acousonde (figure 1*a* and table 1). Record duration varied between 15 and 378 h (mean ± s.d.: 148.5 ± 157.5 h). The depths of prey capture events varied across individuals, ranging from 2 to 641 m (mean ± s.d.: 164.6 ± 157.8 m, figure 1*b* and electronic supplementary material, figure S2).

**Table 1.** Summary of the data recorded for each whale. These individuals refer to ones not published in Heide-Jørgensen *et al*. [9]. ‘Acou’ stands for Acousonde. The Acousonde information presented here only covers the period in common with STP information and may therefore differ from Blackwell *et al*. [8]. The last row refers to the means ± s.d.

ID	sex	tag type	start	end	duration (h)	ndrops	ndaily drops	depths at drops (m)	n buzzes	ndaily buzzes	depth at buzzes (m)
Mara	F	STP	2014-08-11 16:15:00	2014-08-24 21:18:00	317	224	17.0	218.2 ± 256.6	—	—	—
Thora	F	STP + Acou	2014-08-12 01:36:10	2014-08-15 20:32:16	91	21	5.5	283.0 ± 198.2	4193	1106.6	323.7 ± 176.7
7617	F	STP	2015-08-14 16:25:00	2015-08-15 07:03:00	15	4	6.6	7.8 ± 12.9	—	—	—
7618	F	STP	2015-08-14 17:37:00	2015-08-30 11:51:00	378	129	8.2	44.4 ± 95.6	—	—	—
Frida	F	STP + Acou	2015-08-16 21:30:01	2015-08-19 05:11:42	56	75	32.3	39.4 ± 96.0	887	382.2	170.5 ± 135.1
Eistla	F	STP + Acou	2016-08-25 06:30:00	2016-08-26 16:57:10	34	5	3.5	394.7 ± 143.8	907	631.8	316.9 ± 129.5
					148.5 ± 157.5	76.3 ± 87.1	12.2 ± 10.9	164.6 ± 157.8	1995.7 ± 1903.0	706.9 ± 368.0	270.4 ± 86.6

Both the recovery time (range: 1.5–57 min, excluding events lasting greater than 60 min due to data gaps: approx. 3% of the dataset) and the ST drop magnitude (range: 0.6–22.2°C) were variable across events and individuals (electronic supplementary material, figure S3). Some events occurred at depth with a recovery of medium duration (18 min), while others were located much closer to the surface and associated with temperature drops of greater magnitude with a longer recovery (e.g. 29 min, figure 1*c*).

For some whales, buzzes were produced during shallow and short dives that were not associated with prey capture events—referred to as unsuccessful foraging dives (e.g. Thora, figure 1*d*). Although some differences were visible across individuals, the depths of foraging activity (as per buzzes) and prey capture events (as per ST drops) generally agreed when examined for all three whales (figure 1*d*).

### (b) Buzz versus stomach temperature drop frequency

Compared to the number of daily buzzes (range: 382.2–1106.6, mean ± s.d.: 706.9 ± 368), the number of daily prey capture events was low for all whales equipped with an Acousonde (range: 3.5–32.3 per individual, mean ± s.d.: 12.2 ± 10.9, table 1 and electronic supplementary material, figure S4). A small portion of the dives (less than 4%) was associated with ST drops but no buzz.

### (c) Success rate

**Figure 2.**
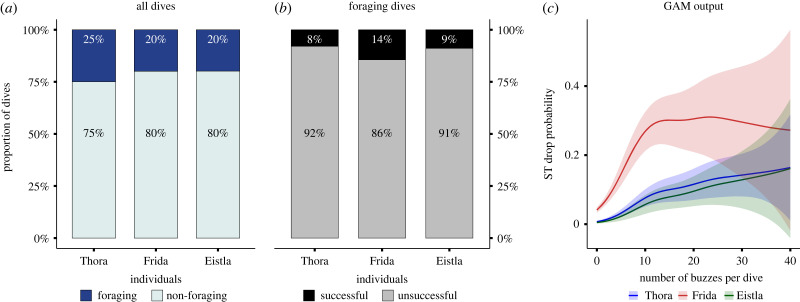
Proportion of non-foraging (no buzz, no ST drop) and foraging (with buzz) dives in the three Acousonde-carrying whales. A dive is defined below 2 m (after zero offset correction) and with duration > 30 s. (*b*) Proportion of successful (buzzes and ST drop) versus unsuccessful dives (buzzes but no ST drop) within the foraging dive category. (*c*) Individual smooth curves derived from the GAM showing the relationship between the probability of ST drop (prey capture event) and the number of buzzes per dive (foraging activity).

Non-foraging dives represented 75–80% of all dives, while 20–25% were dedicated to foraging (through presence of buzzes, figure 2*a*). Among foraging dives, only 8–14% were successful in that they included both buzzes and ST drops, while 86–92% could be deemed unsuccessful, as they included buzzes but no ST drop (figure 2*b*). The probability of a drop increased with the number of buzzes per dive (GAM deviance explained: 19%), but some differences were observed across individuals (figure 2*c*).

### (d) Corrected feeding rate and daily food consumption

The estimated daily food intake was 2.8 ± 1.5 kg day^−1^ during summer, which is in agreement with the low mass of stomach contents collected from narwhals in summer (electronic supplementary material, figure S5). This represents an average daily food consumption of only 0.4% (± s.d.: 0.2) of the body mass of adult narwhals in summer (table 2).

**Table 2.** Corrected daily feeding rates and estimated daily food consumption of narwhals in summer. Individuals with an asterisk refer to published data from Heide-Jørgensen *et al.* [9]*.* The last row refers to the means ± s.d.

ID	sex	year	length (cm)	body mass (kg)	feeding rate (d^−1^)	n drops from STP	n drops from Acousonde	correction factor	n drops corrected	feeding rate corrected (d^−1^)	food intake (kg d^−1^)	food consumption (%biomass d^−1^)
22849	m	2012*	225	546	8	5	—	—	9.8	15.7	2.4	0.4
22850	M	2012*	400	849	2.6	45	—	—	88.3	5.1	0.8	0.1
22853	M	2012*	278	370	4.8	2	—	—	3.9	9.4	1.4	0.4
22638	F	2012*	400	898	14.8	8	—	—	15.7	29	4.3	0.5
3963	M	2013*	400	849	13	13	—	—	25.5	25.5	3.8	0.4
3964	M	2013*	356	629	14.4	18	—	—	35.3	28.2	4.2	0.7
6335	M	2013*	390	793	9.7	19	—	—	37.3	19	2.9	0.4
3965	M	2013*	440	940	11.8	94	—	—	184.4	23.2	3.5	0.4
Mara	F	2014	341	557	17.0	224	—	—	439.5	33.3	5	0.9
Thora	F	2014	390	828	5.5	19	21	1.11	—	—	0.8	0.1
7617	F	2015	400	898	6.6	4	—	—	7.8	12.8	1.9	0.2
7618	F	2015	410	974	8.2	129	—	—	253.1	16.1	2.4	0.2
Frida	F	2015	380	764	32.3	19	75	3.95	—	—	4.8	0.6
Eistla	F	2016	360	650	3.5	6	5	0.83	—	—	0.5	0.1
			369.3 ± 56.6	753.2 ± 176.6	10.9 ± 7.6	43.2 ± 64.0	33.7 ± 36.7	1.96 ± 1.73	100.1 ± 138.8	19.8 ± 8.9	2.8 ± 1.5	0.4 ± 0.2

## 4. Discussion

Despite being critical in understanding the energetic costs and fitness consequences of animals in the wild, information on prey capture efficiency is unknown in the majority of free-ranging marine taxa. The availability of STP signals on the Acousonde recordings provided an opportunity to adjust the satellite-transmitted numbers in a way that better reflects the actual number of feeding events by summering narwhals, also making consecutive drops easily discernible. The comparison of concurrent recordings from both tags revealed that on average the Acousonde provided almost twice as many detections as the STP tags (table 2). The low capture rate was supported by the examination of stomach contents of narwhals during indigenous hunts in the summer [14], showing a low density of prey items (individual masses less than 150 g) in several narwhal populations [9,15–17].

The capture rate after corrections from Acousonde detections (19.8 ± 8.9 ST drops day^−1^) contrasts with the high daily number of buzzes (707 ± 368), confirming a low feeding success rate despite an intense foraging activity. The feeding tactics of narwhals are still poorly understood but they may target schooling prey. The ingestion of several prey at a time makes the detection of feeding events using STPs challenging [18], leading to a possible underestimation of the daily feeding rate in our study. Even though the main prey of narwhals (squid *Gonatus fabricii*) may be found in schools [19], the concurrent use of accelerometers and acoustics in summering narwhals suggested that they target solitary items at depth (likely squid) and more schooling prey in shallower layers (less than 100 m [20]). Although our sample size was small in terms of individuals, showing some inter-individual variability, the success rate was stable across whales (s.d.: 3.5%).

The absence of buzzes during some feeding dives also suggests that the use of buzzes alone to identify foraging attempts in narwhals might be inadequate. Shallow prey capture events could alternatively reflect a distinct behaviour of catching the prey at depth but processing it in shallower water as has been observed in fur seals [21]. Alternatively, ST drops occurring close to the surface could be associated with ingestion of water (mariposa), as water salinity is lowest in the top few metres due to ice melting in the summer. However, marine mammals are known to meet their water requirements primarily from pre-formed water in their diet [22–24], and the elevated water content of narwhal prey (e.g. squid contain 79–84% water [25]), makes water ingestion events unlikely.

Narwhals are known to feed mainly on squid, but also on polar cod (*Boreogadus saida)* and Arctic cod (*Arctogadus glacialis*), and to a lesser extent on Greenland halibut (*Reinhardtius hippoglossoides*), capelin (*Mallotus villosus*) and pelagic crustaceans [9,11]. Based on regression equations for *Gonatus spp.* [26] and polar cod [15], Heide-Jørgensen *et al.* [9] estimated that squid beaks and otoliths from narwhal stomachs came from squid and polar cod with individual masses less than 100 g, which reinforced our assumption of individual prey item ingested less than 150 g. In offshore waters, most of these prey are found in deep waters (200–1200 m [10], while capelin is considered a mid-water species (50–200 m [27]) that seasonally occurs in shallow waters (less than 10 m [28]), suggesting that narwhals may target adult squid and polar cod in deep layers using buzzes, but juvenile squid and capelin at shallower depths using visual cues.

The low feeding rate of narwhals in summer could be a result of low prey density, extreme prey selection or lowered energy needs. A characteristic of Arctic whales is the thick blubber layer (up to 10 cm in narwhals (Greenland Institute of Natural Resources 2010, unpublished data)) that limits heat loss to the environment [29], reduces options for heat dumping during exercise, and can seasonally be mobilized during times of nutritional stress, e.g. migration. Our results suggest that the main period of prey intake and lipid build-up for narwhals takes place in the offshore winter habitats when the whales are not migrating. A seasonal variation in the blubber thickness has been demonstrated in a closely related species, the beluga (*Delphinapterus leucas*), supporting the possibility of blubber deposition for energy storage in winter in narwhals [30]. This emphasizes the critical importance of prey availability of these winter habitats and implies that body reserves gained in winter may to some extent sustain the whales’ activities year-round.

In summer, narwhals are both niche-conservative and cold-adapted with a preferred feeding temperature of less than 2°C [31], yet climate change promotes warmer sea temperatures on their summer grounds [32] as well as the predicted disappearance of their winter habitat [33]. Our findings, therefore, raise concerns about the strong dependence this deep-diving predator has on its winter habitat. The scattered prey field, ongoing ecological changes and increasing anthropogenic disturbance [32,34,35] make the identification of energetically important habitats crucial for Arctic animals.

## Data Availability

The data are available from the Dryad Digital Repository: https://doi.org/10.5061/dryad.15dv41p1f [36]. The data are provided in the electronic supplementary material [37].
